# Zhuanggu Guanjie herbal formula mitigates osteoarthritis *via* the NF-κB transduction mechanism

**DOI:** 10.3389/fphar.2022.896397

**Published:** 2022-12-01

**Authors:** Gong Guowei, Zheng Yuzhong, Zhou Xuan, Dai Zhi, Duan Juanhui, Wang Jing, Yang Peikui, Liu Xiangzhi, Wen Zhen

**Affiliations:** ^1^ Department of Bioengineering, Zunyi Medical University, Zhuhai Campus, Zhuhai, China; ^2^ Guangdong Provincial Key Laboratory of Functional Substances in Medicinal Edible Resources and Healthcare Products, Hanshan Normal University, Chaozhou, China; ^3^ China Resources Sanjiu Medical & Pharmaceutical Co., Ltd., Shenzhen, China; ^4^ Department of Medical Laboratory, Chaozhou People’s Hospital, Chaozhou, China; ^5^ College of Chemistry and Environmental Engineering, Shenzhen University, Shenzhen, China

**Keywords:** Zhuanggu Guanjie capsules, herbal medicine, NF-κB, anti-osteoarthritis, inflammation

## Abstract

The Zhuanggu Guanjie herbal formula has been a famous Chinese prescription for treating bone diseases since time immemorial. The anti-osteoarthritis (OA) properties of this botanical prescription are well documented in the Chinese Pharmacopoeia. However, the detailed mechanisms behind the phenomenon have not been elucidated. Hence, we aimed to investigate the anti-OA efficacy of the Zhuanggu Guanjie herbal formula and its underlying mechanism. The anti-OA properties of Zhuanggu Guanjie capsule (ZGC) were determined by the cytokine contents and inflammatory-related proteins, which were measured by RT-PCR, flow cytometry, Western blot, and laser confocal assay in ATDC5 cells. The levels of interleukin-6, tumor necrosis factor-α, inducible nitric oxide synthase, cyclooxygenase-2, and prostaglandin synthesis E2 have been markedly reduced after being treated with ZGC for 48 h in a dose-dependent manner. Furthermore, ZGC prevented the translocation of NF-κB from the cytosol to the nucleus. On the other hand, we used the mono-iodoacetate (MIA)-induced OA model to confirm the *in vivo* efficacies of this herbal formula. Oral administration of ZGC attenuated MIA-induced OA damage through changes in histopathological and knee joint volumes. The serum matrix metalloproteinase-13 contents in the ZGC treatment group declined as compared to those in the MIA model group. Through our *in vitro* and *in vivo* studies, we confirmed the anti-OA efficacy of ZGC and uncovered its detailed mechanism, and this treatment shed light on OA pathophysiology.

## Introduction

Osteoarthritis (OA) is a classification of degenerative musculoskeletal diseases that regularly cause chronic pain and joint dysfunctions (Felson et al., 2000a; Felson et al., 2000b). This condition is marked by chronic inflammatory responses to the synovial membrane and gradual degradation of articular cartilage (Woodell-May and Sommerfeld, 2020). Damage to cartilage causes synovial inflammation, and chondrocyte-mediated inflammatory responses are a key factor in the development of OA (Scanzello, 2017). The pathophysiology of OA remains a mystery. Numerous studies have shown that inflammatory responses are a critical factor in the progression of this joint degenerative disease (Sokolove and Lepus, 2013). As a result, reducing chondrocyte inflammation is critical for slowing or stopping the progression of OA. Steroids are the standard treatment for OA (de Lange-Brokaar et al., 2012). Long-term treatments with steroids cause various bone diseases such as osteoporosis and joint deformity (Felson et al., 2000a; Felson et al., 2000b). As a result, people would like to use traditional Chinese medicine (TCM) to mitigate OA and improve their life qualities.

TCM classifies OA as arthralgia syndrome, a term derived from the ancient book “Huangdi Neijing.” Previous research has shown that TCM generally prescribes anti-inflammatory and anti-osteoporosis medicine for OA patients (Yang et al., 2017). Among the most commonly used Materia Medica, *Rehmannia glutinosa* Libosch. [Rehmanniae Radix, RR] and *Psoralea corylifolia* L. [Psoraleae Fructus, PF] were first recorded in “Shennong Bencao Jing” (Gong et al., 2019; Ota et al., 2019). According to Chen et al. (2014), RR was the most commonly used herbal remedy for the treatment of musculoskeletal degenerative disease. Jhun et al. (2018) discovered that RR extracts reduced cartilage degeneration in OA rat model joints by suppressing matrix metalloproteinase-13 (MMP-13) and nitrotyrosine. Siu et al. (2019) reported that PF extracts inhibited inflammatory mediator release *via* the PI3K/Akt pathway *in vitro*. After 14 days of oral administration of aqueous extracts of PF on the OA rat model, plasma levels of interleukin (IL)-6, tumor necrosis factor-α (TNF-α), and inducible nitric oxide synthase (iNOS) were significantly decreased as compared with those in the sham group (Siu et al., 2019).

A variety of transduction pathways have been identified to serve as necessary features in monitoring the progression of OA, especially the nuclear factor kappa-light-chain-enhancer of the activated B cell (NF-κB) pathway (Gong et al., 2018, 2020). In the cytosol, NF-κB interacts with inhibitory kappa-B (IκBα) under normal conditions (Gong et al., 2017). After receiving a stimulus, IκBα kinase is activated by phosphorylating IκBα protein, then stimulating translocation of NF-κB from the cytosol to the nucleus, and triggering the expression of target genes such as IL-1β, IL-6, TNF-α, iNOS, cyclooxygenase-2 (COX-2), and prostaglandin synthesis E2 (PGES-2) (Gong et al., 2020). The production of cytokines or inflammatory mediators significantly accelerates the breakdown of the extracellular matrix, resulting in the aggravation of OA progression (Geyer and Schönfeld, 2018; Ansari et al., 2020).

Zhuanggu Guanjie capsule (ZGC) contains 12 Materia Medica, and they are *Cibotium barometz* (L.) [Rhizoma Cibotii], *Epimedium brevicornum* Maxim. [Epimedii Folium], *Angelica pubescens* Maxim. f. biserrata Shan et Yuan [Angelicae Pubescentis Radix], *Drynaria fortunei* (Kunze) J. Sm. [Rhizoma Drynariae], *Dipsacus asper* Wall. ex Henry [Dipsaci Radix], *Psoralea corylifolia* L. [Psoraleae Fructus], *Taxillus chinensis* (DC.) Danser [Taxilli Herba], *Spatholobus suberectus* Dunn [Spatholobi Caulis], *Rehmanniia glutinosa* Libosch. [Rehmanniae Radix], *Aucklandia lappa* Decne. [Aucklandiae Radix], *Boswellia carteri* Birdw. [Olibanum], and *Commiphora myrrha* Engl. [Myrrha] at the ratio of 5: 3: 3: 4: 5: 8: 5: 3: 9: 3: 3: 3. In fact, RR and PF are the predominant botanical drugs. It has been proposed in the Chinese Pharmacopoeia (CP, 2020) as a treatment for OA patients [18], but the precise pharmacological mechanisms of the ZGC remain unknown. Hence, in this study, we aimed to examine the transcriptional activity of this formula on cultured ATDC5 cells and mono-iodoacetate (MIA)-induced OA animal models.

## Methods and materials

### Preparation of Zhuanggu Guanjie capsule extract

ZGC was purchased from China Resources Sanjiu Medical & Pharmaceutical Co., Ltd., and the catalog number was 1912002S. The mixture was resolved in water by sonication (Dong et al., 2006). The extracts were dried by lyophilization and stored at −80°C at the ultimate concentration of 100 mg/ml.

### RRLC-QQQ-MS/MS condition

The quantification analysis was performed by RRLC-QQQ-MS/MS. For liquid chromatography analysis, an Agilent RRLC 1200 series system (Agilent) equipped with a degasser, a binary pump, an auto-sampler, a DAD, and a thermostatted column compartment was used. A volume of 2 μl (after passing through a 0.45-μm Millipore filter) of samples was injected. The herbal extract was separated on an Agilent ZORBAX SB-C18 column (1.8 µm, 4.6 × 50 mm). The mobile phase was composed of 0.1% formic acid in acetonitrile (A) and 0.1% formic acid in water (B). A linear gradient elution was applied from 15–20% B at 0–3 min, 20–30% B at 3–8 min, 30–40% B at 8–14 min, 40–75% B at 14–18 min, and 75–80% B at 18–20 min. For the MS/MS analysis, an Agilent QQQ-MS/MS (6410A) equipped with an electrospray ionization (ESI) ion source was used. Two suitable transition pairs were chosen for acquisition in multiple reaction monitoring (MRM). The ionization source conditions were as follows: the flow rate was 0.3 ml/min; the column temperature was 25°C; the drying gas temperature was 325°C; drying gas flow: 10 L/min; nebulizer pressure: 35 psig; capillary voltage: 4,000 V; and delta electro multiplier voltage: 400 V. The fragmentor voltage and collision energy values were optimized to obtain the highest abundance.

### Cell culture

ATDC5 cells were purchased and shipped from the American Type Culture Collection (ATCC, Manassas, VA) and grown in the DMEM/F12 medium containing 100 IU/ml penicillin, 100 μg/ml streptomycin, and 10% fetal bovine serum. ATDC5 was incubated in 37°C incubators with 5% CO_2_. Once the cells were 70% confluent, the medium was supplemented with 1% ITS to induce chondrocyte-like cells. Chondrocyte-like cells were pretreated with lipopolysaccharide (LPS, 1 μg/ml) for 24 h before drug or herbal extract presence. LPS was obtained from Sigma (St. Louis, MO).

### Propidium iodide-Annexin staining assay

Cells were seeded in 35-mm culture plates and allowed to grow for 24 h before drug treatment. The Annexin V-FITC/PI Detection Kit was employed for flow cytometry (BD Biosciences, Franklin Lakes, NJ). Briefly, cells were washed twice with PBS and then harvested. Cells were incubated in 100 μl of binding buffer, containing Annexin-V/FITC and propidium iodide (PI), for 15 min at room temperature and kept in the dark. The samples were automatically acquired using the loader with acquisition criteria of 10,000 events for each tube, and the quadrants were set according to the population of samples. The results were analyzed using FlowJo v10.6 software.

### Real-time PCR

The transcriptional levels of IL-1β, IL-6, and TNF-α were detected in LPS-stimulated chondrocyte-like cells. After ZGC treatment for 48 h, total RNA was isolated by RNAzol reagent (Molecular Research Center, Cincinnati, OH) and then reverse-transcribed into cDNAs by employing M-MLV (Moloney murine leukemia virus) reverse transcriptase according to the manufacturer’s suggestions (Sigma). Real-time PCR was employed here by using FastStart Universal SYBR Green Master (ROX) according to the manufacturer’s instructions (Roche Applied Science, Mannheim, Germany). The sequences of primers were shown as follows: 5′-AAA TAC CTG TGG CCT TG-3′ (sense primer, S) and 5′-TTA GGA AGA CAC GGA TTC-3′ (antisense primer, AS) for murine IL-1β (NM_008361); 5′-GGA GTA CCA TAG CTACCT GG-3′ (S) and 5′-CTA GGT TTG CCG AGT AGA TC-3′ (AS) for murine IL-6 (NM_031168); 5′- AGT GAC AAG CCT GTA GCC -3′ (S) and 5′-AGG TTG ACT TTC TCC TGG-3′ (AS) for murine TNF-α (NM_013693); 5′-GAT GAG GCT GCG GAA GAA GG-3’ (S) and 5′-GCG AAG GCG TGG GTT CAG-3’ (NM_022415). Glyceraldehyde 3-phosphate dehydrogenase (GAPDH) was used as an internal control, and the sequences were shown as follows: 5′-AAC GGA TTT GGC CGT ATT GG-3′ (S) and 5′-CTT CCC GTT CAG CTC TGG G-3′(AS) (NR_0215885). The SYBR green signal was detected using the ABI 7500 Fast Real-Time PCR system (Applied Biosystems, Foster City, CA). Transcript levels were quantified by applying the ΔCt value method (Dong et al., 2006; Gong et al., 2016), where the values of target genes were normalized by GAPDH in the same sample. Dexamethasone (Dex, 10 μM) was obtained from Sigma and used as a positive control in all experiments.

### Protein expression of cytokines

Protein expression of pro-inflammatory cytokines after drug treatment for 2 days was revealed by MILLIPLEX^®^ technology (Millipore, MA). Supernatants of the culture medium were collected to measure the concentrations of cytokines (IL-1β, IL-6, and TNFα) using MILLIPLEX^®^ technology, following the manufacturer’s instructions.

### Reactive oxygen species formation

Fluorometric measurements of reactive oxygen species (ROS) were performed using the CellROX Deep Red Fluorescence Flow Cytometry Detection Kit (BD Biosciences) under the manufacturer’s suggested protocol. Briefly, cells were incubated in 100 μl of binding buffer for 15 min at room temperature. Samples were automatically acquired using the loader with acquisition criteria of 10,000 events for each tube, and the quadrants were set according to the population of viable tubes. The cytometry results were analyzed using FlowJo v7.6 software.

### Western blot

The cell lysate was subjected to SDS-PAGE to reveal the protein expression levels of target genes. After transferring the target proteins to membranes, the membranes were incubated with anti-iNOS (CST, Danvers, MA) at 1: 5,000 dilutions, anti-COX-2 (CST) at 1: 5,000 dilutions, anti-PGES2 (Santa Cruz, Dallas, TX) at 1:5,000 dilutions, and anti-GAPDH (Santa Cruz) at 1: 5,000,000 dilutions at 4°C for overnight. Following incubation in horseradish peroxidase (HRP)-conjugated secondary antibodies for 3 h at room temperature, the immune complexes were visualized using the enhanced chemiluminescence (ECL) method (Amersham Biosciences, Piscataway, NJ). The band intensities were calculated and analyzed using Image J.

### Laser confocal assay

The translocation of the NF-κB p65 subunit was activated by treatment with 1 μg/ml of LPS for 24 h and then presented with ZGC for another 48 h. Then, cells were fixed for 10 min in 4% methanol-free paraformaldehyde. Antibodies were diluted in PBS containing 2.5% fetal bovine serum and 0.1% Triton X-100 (Sigma). The NF-κB p65 subunit antibody was diluted at 1:500 in PBS. The samples were incubated overnight in a cold room. Then, 1: 1,000 dilution of the secondary FITC-conjugated F(ab’)2 fragment donkey anti-rabbit IgG antibody (Jackson Laboratories, West Grove, PA) was added and incubated at room temperature for 3 h in the dark. After incubation, the cells were washed three times with PBS. Before performing the assay, a 1:5,000 dilution of DAPI was added to the nucleus. Fluorimetric measurements were performed using an Olympus FluoView FV1000 laser scanning confocal system (Olympus America, Melville, NY) mounted on an inverted Olympus microscope equipped with a 63× objective.

## Animals

Wistar rats weighing approximately 400–450 g were maintained at 21 ± 2°C with food and water. The experimental procedures were approved by the Animal Experimentation Ethics Committee of Zunyi Medical University (Animal Ethics Approval No. ZMU21-2022-071) and were conducted in accordance with the guidelines of “Principles of Laboratory Animal Care.”

### Mono-iodoacetate-induced osteoarthritis animal models

A total of 36 male Wistar rats were randomly divided into six groups as follows: control, the model group, the dexamethasone (Dex) group, and a series dosage of ZGC groups. On days 1 and 3, male Wistar rats were injected with 100 μl of 100 mg/ml MIA (Sigma, St. Louis, MO, United States) into the left knee junction. From day 7 until the completion of the experiment, the mice were treated with Dex (10 mg/kg) or different dosages of ZGC (12.5, 25, and 50 mg/kg, which indicated ZGC-L, -M, and -H, respectively) for another 14 days *via* gavage every other day, then blood samples were collected, and histopathological analysis was performed.

### Joint volume measurement

The change in the joint volume was measured twice using a plethysmometer (Ugo Basile, Comerio VA, Italy). The first measurement was carried out on day 0, and the reading was set as the baseline. The second measurement was performed after drug treatment for 14 days.

### Histological staining

The joint tissue was subsequently fixed in 10% formaldehyde and embedded in paraffin. Blocks were cut into 5-μm-thick slices for hematoxylin and eosin (H&E) or MMP-13 staining.

### Serum matrix metalloproteinase-13 levels

Blood samples were collected from six animal groups after drug treatment for 14 days. The total MMP-13 level in serum was measured using an ELISA kit following the manufacturer’s instructions.

### Statistical analysis and other assays

Protein concentrations were measured by Bradford’s method (Hercules, CA). Statistical tests were carried out using a one-way analysis of variance. Data were expressed as mean ± SEM. Statistically significant changes were classified as significant (*) when *p* < 0.05, more significant (**) when *p* < 0.01, and highly significant (***) when *p* < 0.001 as compared with the control group and significant (^) when *p* < 0.05, more significant (^^) when *p* < 0.01, and highly significant (^^^) when *p* < 0.001 as compared with the inflammation group.

## Results

**FIGURE 1:**
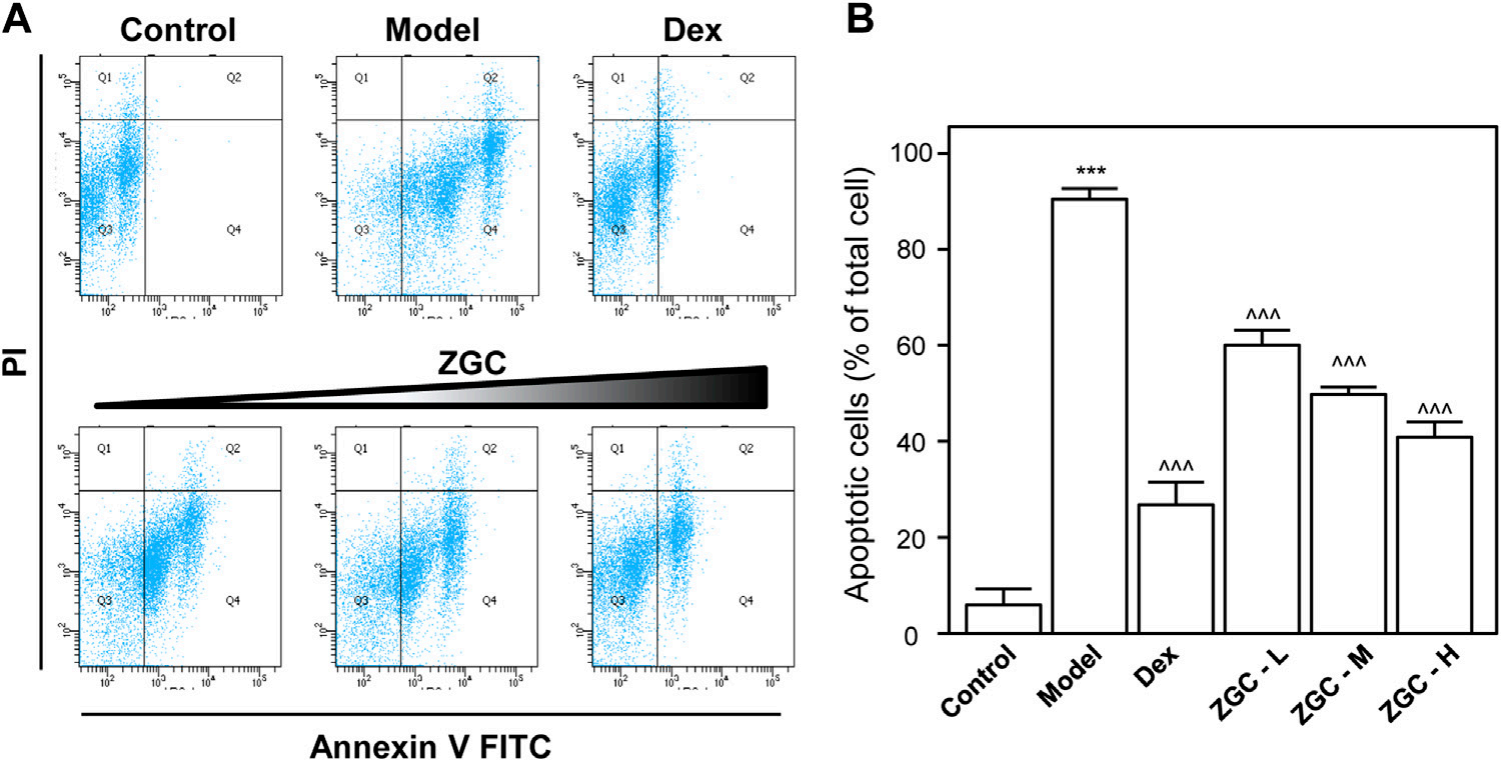
ZGC mitigates cell apoptosis. Cultured cells were treated with LPS (1 μg/ml) for 24 h and then incubated with a series of ZGC (0.125, 0.25, and 0.5 mg/ml) for another 48 h. Dex (10 μM) was used as the positive control. **(A)** Dual parametric dot plots combining Annexin V-FITC and PI fluorescence showed the viable cell population in the bottom left quadrant (Q3), the early apoptotic cells in the bottom right quadrant (Q4), and the late apoptotic cells in the top right quadrant (Q2). **(B)** Quantification analysis was performed by FlowJo v7.6 software. The values are expressed as the percentage of the total cell number, *n* = 3, in mean ± SEM. ***p < 0.001 was considered a significant result compared to the control group. ^^^*p* < 0.001 was categorized as a significant result compared to the model (LPS) group.

The promising approach to treating OA is the stimulation of chondrogenesis (Chien et al., 2020). The developed ATDC5 cells are the most commonly used *in vitro* model for cell research during chondrogenesis (Fan et al., 2018; Li et al., 2019). One of the key indexes representing the OA process is cell viability (Chien et al., 2020). In this study, we used LPS to activate differentiated ATDC5 cells to mimic inflammatory conditions *in vitro*. In the presence or absence of herbal formula, the apoptosis rate was investigated using Annexin V-FITC kit and the PI kit (Figure 1). In the presence of 1 μg/ml LPS, ATDC5 cell viability was drastically reduced (Figure 1). In contrast to the inflammatory conditional group, we discovered that this herbal formula reduced cell apoptosis in a dose-dependent manner (Figure 1). The authentic ZGC herbal extract chromatogram was verified by RRLC-QQQ-MS/MS as shown in Supplementary Figure S1A. The maximal dosage for ZGC utilized for the following experiment was determined by MTT, and we found the maximal concentration to be 0.5 mg/ml (Supplementary Figure S1B).

**FIGURE 2:**
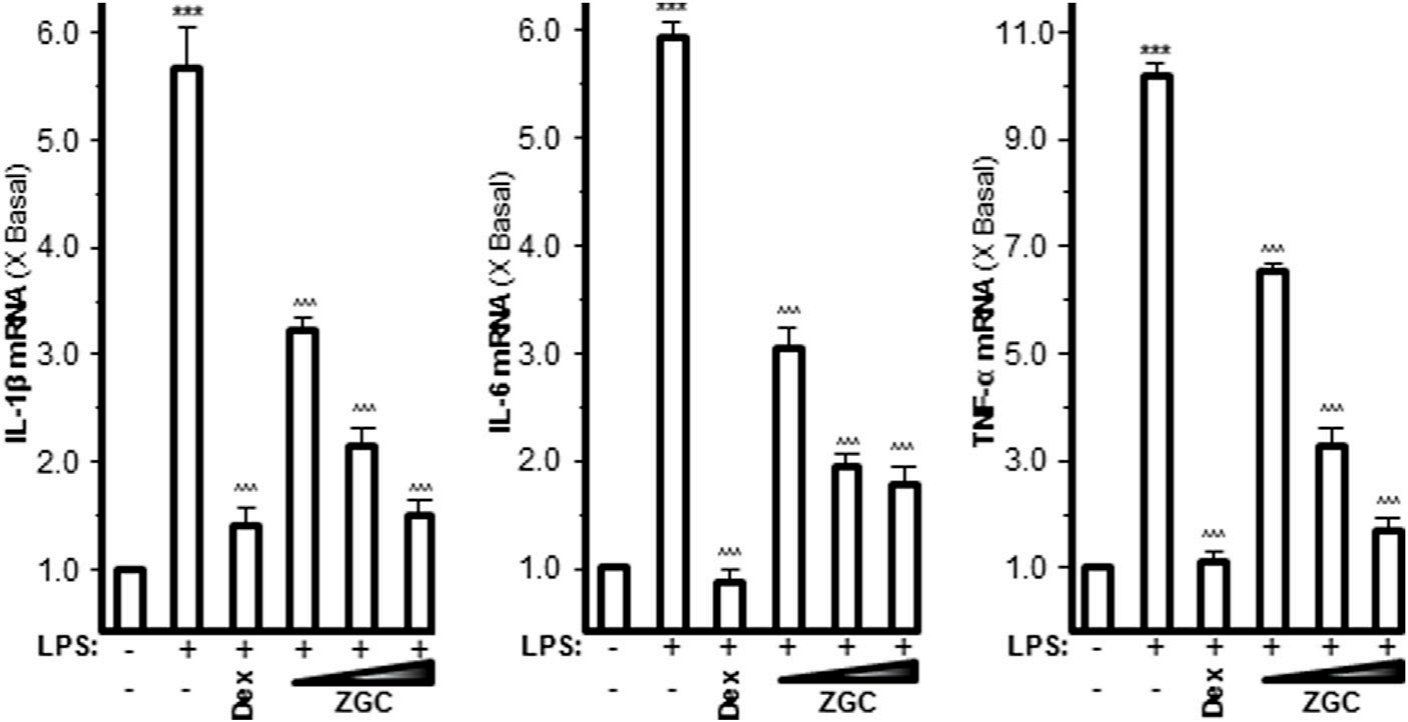
ZGC attenuates mRNA levels of cytokines in cultured LPS-pretreated chondrocyte-like cells. LPS-induced cultured chondrocyte-like cells were incubated with ZGC extracts at different concentrations (0.125, 0.25, and 0.5 mg/ml) for 48 h. The mRNA levels of IL-1β, IL-6, and TNF-α were measured by real-time PCR. GAPDH served as the internal control. Dex (10 μM) was used as the positive control. Data are expressed as the fold of change to the reference reading (blank control group) and as mean ± SEM, where *n* = 3. Statistically significant changes were clustered as highly significant (***) and/or (^^^) when *p* < 0.001 as compared with the control group and/or the LPS-mimicking inflammatory group, respectively.

**FIGURE 3:**
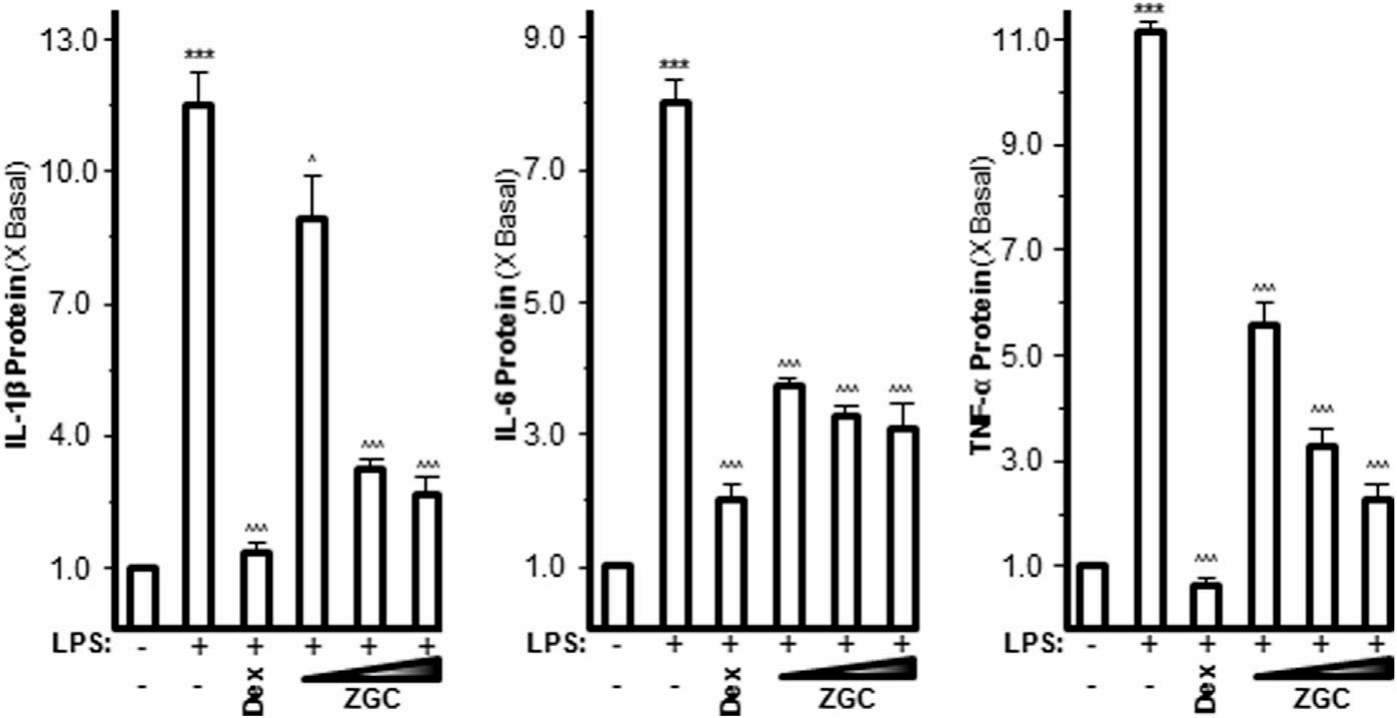
ZGC attenuates cytokine production contents in cultured LPS-pretreated chondrocyte-like cells. LPS-induced cultured chondrocyte-like cells were incubated with ZGC extracts at different concentrations (0.125, 0.25, and 0.5 mg/ml) for 48 h. The cytokine release contents of IL-1β, IL-6, and TNF-α were measured by MILLIPLEX^®^ technology according to standard protocols. Here, Dex (10 μM) was used as the positive control. Data are expressed as the fold of change to the reference reading (blank control group) and as mean ± SEM, where *n* = 3. Statistically significant changes were clustered as highly significant (***) and/or (^^^) when *p* < 0.001 as compared with the control group and/or the inflammatory model group, respectively.

Pro-inflammatory cytokines such as IL-1β, IL-6, and TNF-α are believed to enhance OA progression by creating an inflammatory environment (Sokolove and Lepus, 2013; Yang et al., 2017). LPS-induced upregulation of mRNAs expressing IL-1β, IL-6, and TNF-α was inhibited in a dose-dependent manner by ZGC extract, as revealed by PCR (Figure 2). The maximum suppression was found at 0.5 mg/ml, with 42% suppression for IL-1β, 57% suppression for IL-6, and 73% suppression for TNF-α (Figure 2). Dex was used as a positive control, and it significantly reduced cytokine expression, as shown in Figure 2. The protein levels of these pro-inflammatory cytokines were also reduced after ZGC treatment over 2 days (Figure 3). The alleviation properties of ZGC were dose-dependent, and the maximal concentration was achieved at 0.5 mg/ml (Figure 3).

**FIGURE 4:**
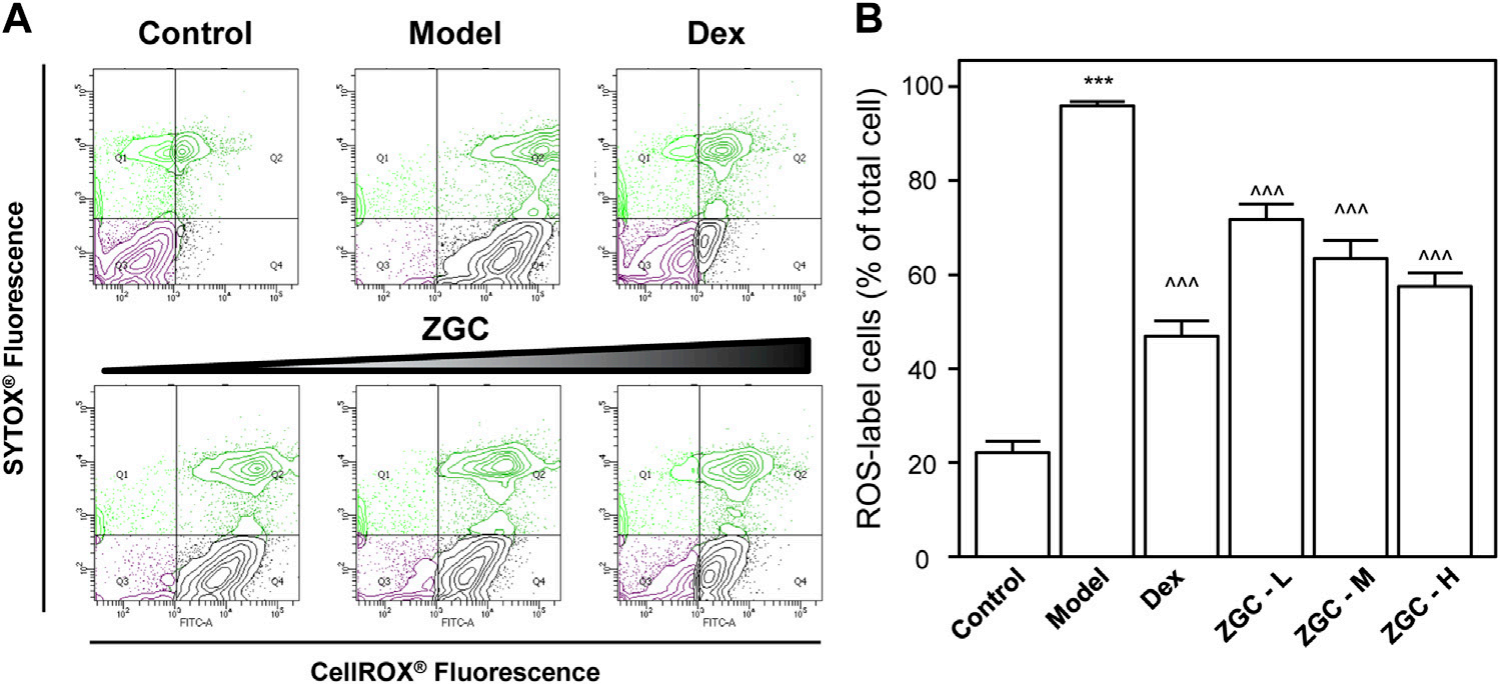
ZGC alleviates ROS formation in LPS-pretreated, differentiated ATDC5 cells. Cultured differentiated ATDC5 cells were pretreated with LPS (1 μg/ml) for 1 day and then incubated with different concentrations of ZGC water extract (0.125, 0.25, and 0.5 mg/ml) or 10 μM Dex for another 48 h **(A)** Fluorimetric measurement by the CellROX^®^ Detection Kit and **(B)** analyzed using FlowJo software. The values are expressed as the percentage of the total cell number, *n* = 3, in mean ± SEM. ***p < 0.001 was considered a significant result compared to the control group. ^^^*p* < 0.001 was categorized as a significant result compared to the model (LPS) group.

**FIGURE 5:**
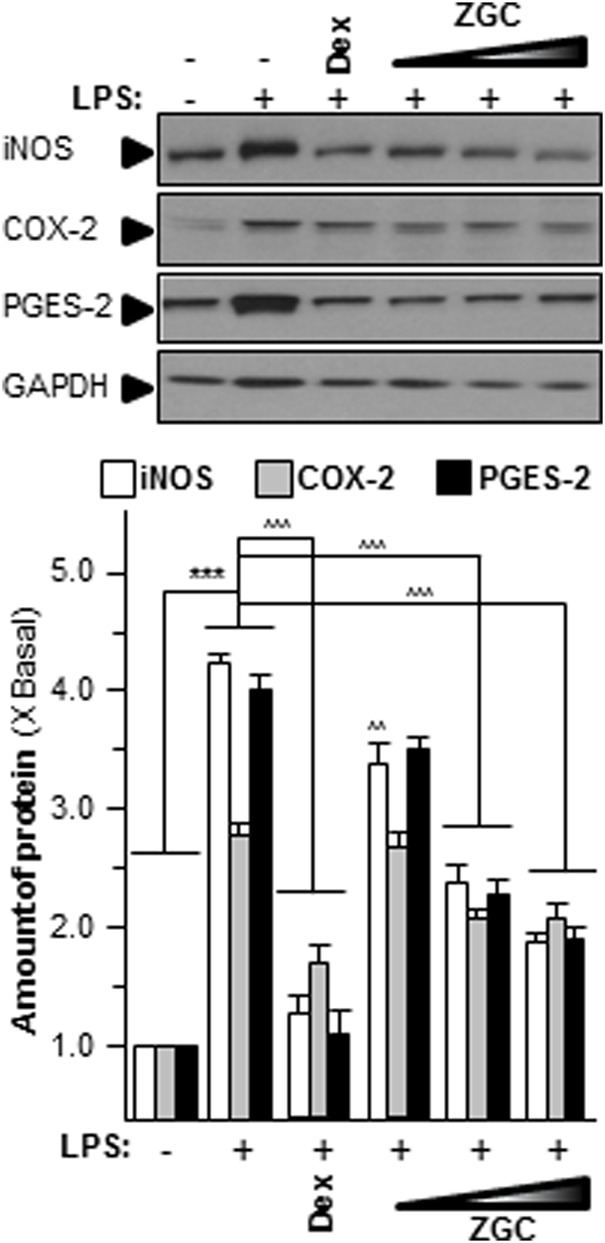
ZGC mitigates iNOS, COX-2, and PGES-2 translational levels *in vitro*. LPS-induced cultured chondrocyte-like cells were incubated with ZGC extracts at different concentrations (0.125, 0.25, and 0.5 mg/ml) for 48 h. Total protein was collected and revealed using immunoblot analysis by specific antibodies. GAPDH served as the internal control; 1 μg/ml of LPS was employed here as an OA model. Dex (10 μM) was used as the positive control. Data are expressed as the fold of change to the reference reading (blank control group) and as mean ± SEM, where *n* = 3. Statistically significant changes were clustered as highly significant (***) or (^^^) when *p* < 0.001 as compared with the blank control group or model group, respectively.

Oxidants have been proven to generate oxidative stress in cells, produce reactive oxygen species (ROS), and subsequently activate tyrosine protein kinases (Woodell-May and Sommerfeld, 2020). ROS affects cell proliferation, differentiation, growth, metabolism, survival, and other functions through protein tyrosine phosphorylation. In addition, ROS activates IκBα kinase, causes IκBα phosphorylation, and ultimately causes cell death (Felson et al., 2000a; Felson et al., 2000b). The contents of these mediators were discovered in LPS-stimulated chondrocyte-like cells. The concentration of ROS was significantly higher in the LPS-only group, which was also the model group (Figure 4). LPS-induced ROS production and generation were considerably reduced when cells were incubated with ZGC (Figure 4). In comparison to the control, the translational characteristics of LPS-induced COX-2, iNOS, and PGES-2 were reduced when different doses of ZGC extract were applied (Figure 5). After 48 h of incubation with ZGC, these suppression functions were found to be dose-dependent but with statistically significant variations (Figure 5). With the addition of 0.5 mg/ml ZGC formula extract, the maximum suppression was achieved (Figure 5).

**FIGURE 6:**
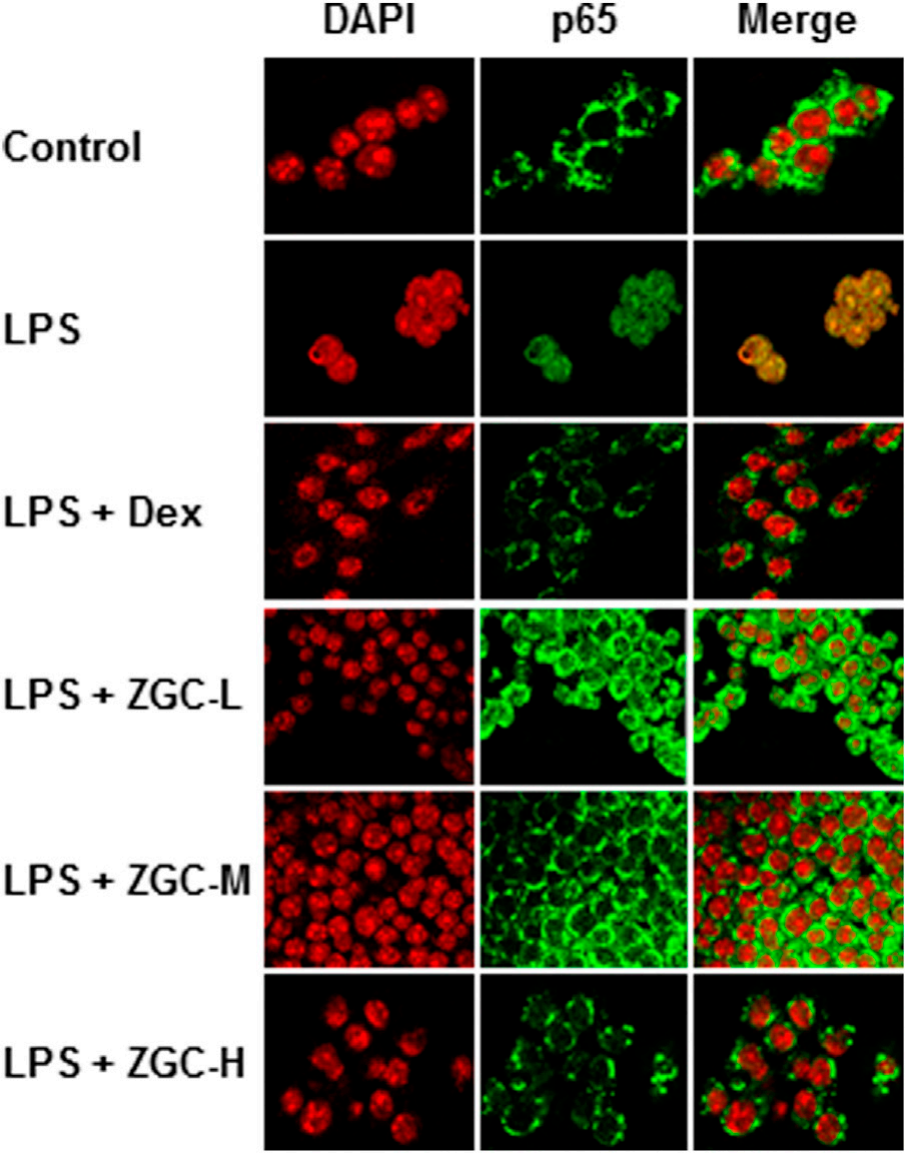
ZGC constricts NF-кB translocation in LPS-pretreated chondrocyte-like cells. The NF-кB p65 translocation properties under the application of ZGC for 48 h were detected in LPS-pretreated chondrocyte-like cells using a confocal microscope. After being treated with a series dosage of ZGC extract (0.125, 0.25, and 0.5 mg/ml) for 48 h, p65 nuclear localization was revealed by confocal microscopy. Nuclei were stained with DAPI. LPS (1 μg/ml) mimicked the inflammation status *in vitro*. Dex (10 μM) was used as the positive control.

When NF-κB is activated, it can translocate into the nucleus, where it regulates the transcription of inflammatory mediators (Woodell-May and Sommerfeld, 2020). LPS induced NF-κB translocation from the cytosol to the nucleus in cultured chondrocyte-like cells, as evidenced by fluorescence co-localization data (Figure 6). As indicated in Figure 6, the translocation functions of this transcriptional factor were reduced after ZGC treatment. After 48 h, Dex effectively inhibited NF-κB translocation from the cytosol to the nucleus (Figure 6).

**FIGURE 7:**
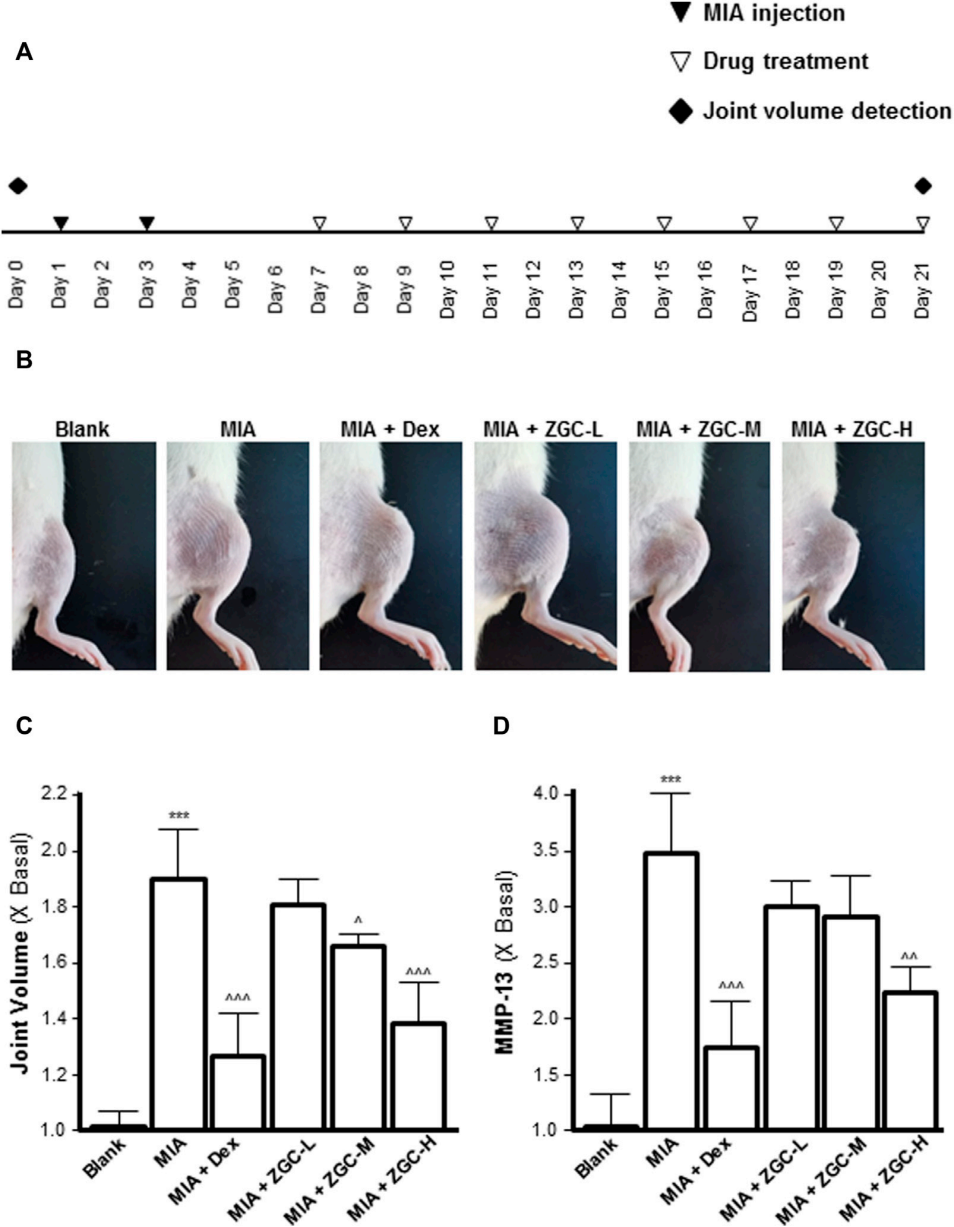
ZGC attenuates MIA-induced OA in the rat model. **(A)** Experimental and knee joint volume detection schedule and the MIA-induced osteoarthritis model. **(B)** Photographic images of the knee joints of different groups of animals. **(C)** Joint edema volume was detected after ZGC treatment for 14 days. **(D)** Blood samples were collected from experimental animals, and the levels of serum MMP-13 were determined using an ELISA kit. Statistically significant changes were classified as highly significant (***) when *p* < 0.001 as compared with the control group and significant (^) when *p* < 0.05, more significant (^^) when *p* < 0.01, and highly significant (^^^) when *p* < 0.001 as compared with the MIC-only group.

**FIGURE 8:**
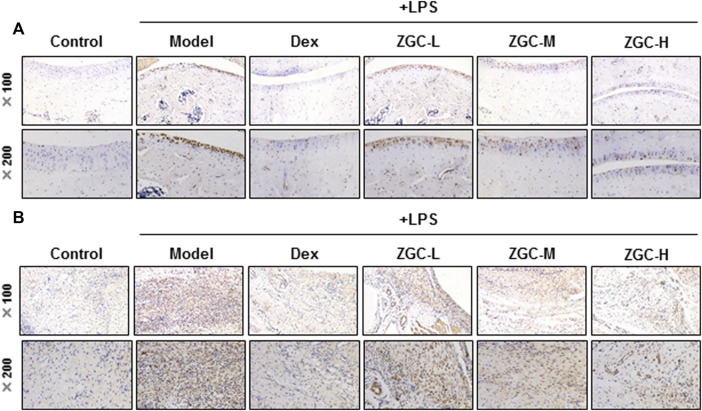
MMP-13 immunohistochemistry analysis of cartilage and synovium. Sections of **(A)** cartilage and **(B)** synovium were stained by the MMP-13 antibody using the immunohistochemistry method after drug treatment (*n* = 6).

**FIGURE 9:**
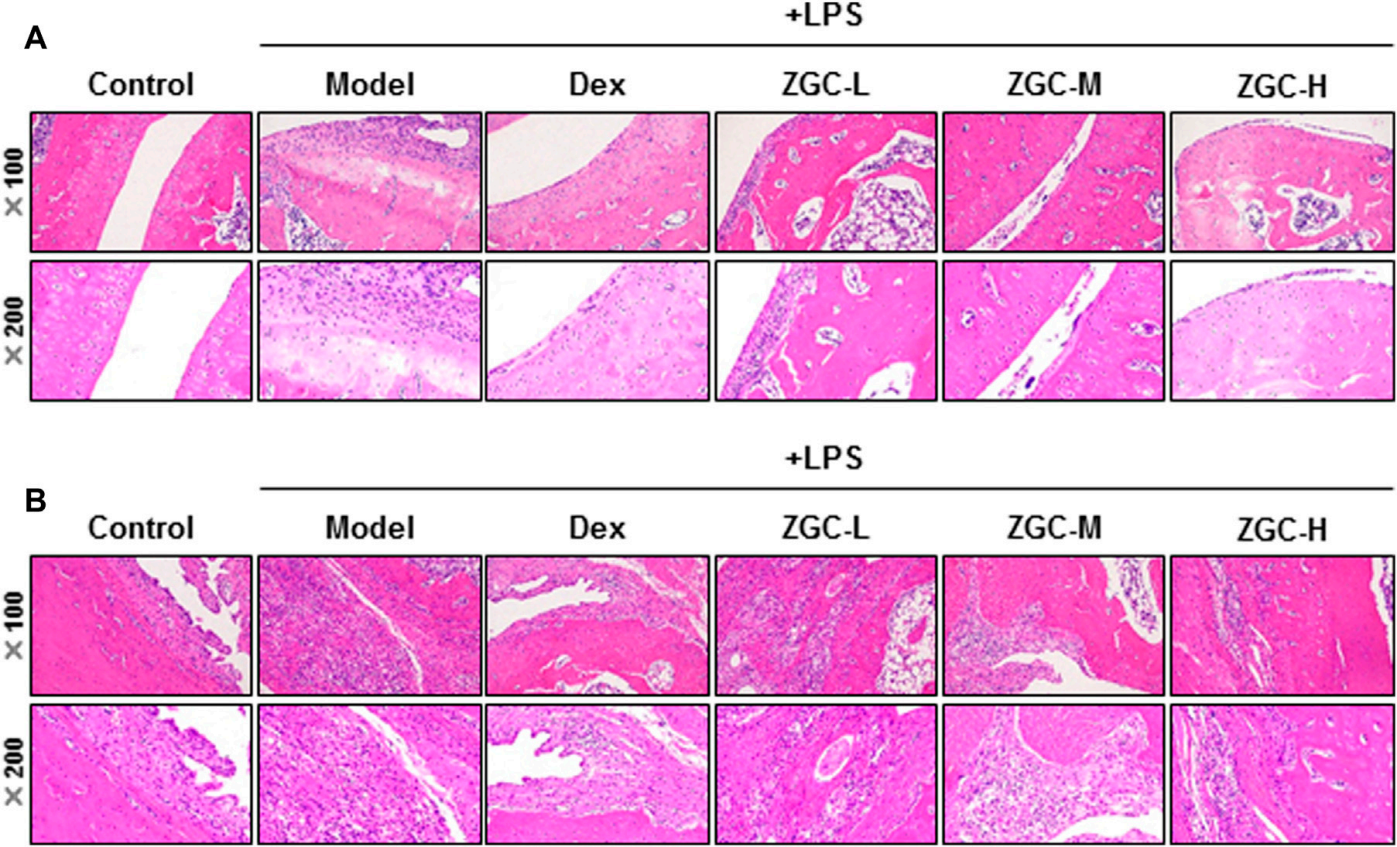
Histological analysis of cartilage and synovium. Sections of **(A)** cartilage and **(B)** synovium were stained by H&E staining after drug treatment for 14 days (*n* = 6).

An MIA-induced OA rat model was used here (Figure 7A). Before injecting MIA, the volume of the knee joint was measured, and the reading served as a baseline. In MIA-induced OA animal models, the knee joint volume was measured after 14 days of herbal medicine treatment (Figures 7B, C). We found that the joint in the MIA group swelled significantly more than that in the blank control group (Figures 7B, C). The joint swelling index was considerably reduced after 2 weeks of ZGC administration, and the higher dose of ZGC (50 mg/kg) suppressed knee edema more effectively than the lower concentration (12.5 mg/kg) (Figures 7B, C). Additionally, serum MMP-13 levels in MIA-induced OA models were measured. MMP-13 production was considerably lower in ZGC-treated rats than in MIA-only rats (Figure 7D). Furthermore, the immunohistochemistry staining of MMP-13 on cartilage and synovium was also presented here within different groups (Figure 8). The results showed that the MMP-13 level was enhanced in MIA-only groups both on cartilage and synovium (Figure 8). Dex was capable of suppressing the MMP-13 content after 14 days of treatment (Figure 8). Moreover, ZGC significantly reduced the MMP-13 expression level in a dose-dependent manner (Figure 8). Representative histological sections, on the other hand, revealed that the ZGC-treated joints exhibited less cartilage and synovium damage than the MIA-only treatment group (Figures 9A, B). The protective effects were also more noticeable in the 50 mg/kg ZGC group than in the 25 mg/kg and 12.5 mg/kg ZGC groups (Figure 9).

## Discussion

TCM has a wide range of materials that are widely used under the TCM principle for disease prevention, diagnosis, and treatment, as well as for regulating the human body. Herbs, animal medications, minerals, magnets, and other items are used in TCM. Herbal medicine makes up the majority of TCM and is widely practiced in our daily lives. According to the “four nature’s” hypothesis, TCM can be divided into four categories: hot, warm, cold, neutral, and aromatic. TCM also appreciates “five flavors,” which are sour, bitter, sweet, spicy, and salty, respectively (Jiang et al., 2020). Each herb experiences its own unique nature and flavor, which account for the herbal properties that influence the Yin–Yang of the body and the prevention and treatment of diseases (Yang et al., 2012).

According to the compatibility of TCM, the role of each herb in a formula can be described as “master, minister, assistant, and servant.” In brief, “master” herbs target the main syndrome of a disease. “Minister” herbs help to improve the therapeutic effect, while “master” herbs reduce the toxicity. “Assistant” herbs cooperate with “master” and “minister” herbs to enhance their therapeutic effects or reverse their accompanying symptoms. “Servant” herbs regulate the properties of other herbs in the herbal mixture. The compatibility of herbs is usually modified in terms of syndrome differentiation (Su et al., 2016; Zhang et al., 2019). In TCM, the herbal mixture is frequently prepared using water and moderate heating, which is a typical and widely used procedure in clinical practice. The extraction procedure has the advantage of being simple to prepare and administer (Zhang et al., 2018). However, the high volume, quality control, and storage challenges are all disadvantages. For ease of transport and storage, herbal formulas are frequently packaged as pills or pulverized powder.

Cytokines and ROS play essential roles in the progression of OA by contributing to bone cell injury and/or death, joint synovial inflammation, chondrocyte anabolism, and cartilage degradation (Du et al., 2013, 2014). IL-1β and IL-6 have been demonstrated to play critical roles in controlling the OA pathological process (Malemud, 2019). These cytokines increase the production of immune-specific mediators such as iNOS, COX-2, and PGES-2. TNF-α is another essential cytokine that plays a role in the development and progression of OA (Quartana et al., 2015). TNF-α increases the expression levels of MMP and “aggrecanase,” a disintegrin and metalloproteinase with thrombospondin motifs (ADAMTSs) (Lieberthal et al., 2015; Malemud, 2019). ROS functions as a second messenger, activating the NF-κB transduction pathway, which results in the creation of inflammatory mediator factors in a cascade (Quartana et al., 2015). These variables cause immature osteoclasts to erode bone, disrupting chondrocyte metabolism and encouraging cartilage deterioration, resulting in permanent fibrosis and exacerbating OA (Lieberthal et al., 2015; Quartana et al., 2015). As a result, reducing cytokine and ROS production is an effective treatment for OA.

RR and PF were widely utilized in increasing renal function and preventing bone disorders according to “Shennong Bencao Jing.” Flavonoids are the active ingredients derived from these two Materia Medica, and they are multi-functional phytochemicals with significant properties that can be used to develop therapeutic medicines for a variety of ailments (Serafini et al., 2010). Flavonoid-enriched RR extract showed neuronal protective effects *via* inhibiting the NF-κB and MAPK signaling pathways according to Xiao et al. (2021). Xie and Du (2011) published similar results, and they found that RR methanol extraction had therapeutic functions for inflammatory treatment by blocking TNF-α, IL-1β, IL-6, and IL-15 production. Jin et al. (2014) reported that oral administration of PF significantly reduced airway hyperresponsiveness to aerosolized methacholine and decreased IL-4 and IL-13 levels in the bronchoalveolar lavage fluid. *In vitro* studies showed that PF ethanol extract suppresses Th2 cytokines such as IL-4, IL-5, and IL-13 in ConA-stimulated D10 cells without affecting their viability (Jin et al., 2014).

The “master” Materia Medica included within this ancient TCM formula are RR and PF, which contain 12 herbal components. We discovered that ZGC was capable of attenuating IL-1β, IL-6, and TNF-α production *via* the NF-κB functioning mechanism based on the present findings, and this treatment shed light on OA pathophysiology. Animal research is still warranted to explore various bone-associated diseases and other pharmacological effects of this traditional herbal compound.

## Data Availability

The original contributions presented in the study are included in the article/Supplementary Material; further inquiries can be directed to the corresponding authors.

## Supplementary material

Supplementary Figure S1 Chromatogram and MTT data of ZGC. **(A)** Typical RRLC-QQQ-MS/MS chromatograms of ZGC are shown here, and the injection volume was 2 μl. **(B)** Cultured cells were treated with a series of ZGC for 48 h. The cell viability was determined by MTT assay. Data are expressed as the percentage of change compared to the control and as the mean ± SEM, where *n* = 3. Statistical changes were clustered as more significant (**) when *p* < 0.01 as compared with the blank group.

## Abbreviation

ADAMTSs, “aggrecanase,” a disintegrin and metalloproteinase with thrombospondin motifs; COX-2, cyclooxygenase-2; CP, Chinese Pharmacopoeia; ECL, enhanced chemiluminescence; H&E, hematoxylin and eosin; HRP, horseradish peroxidase; IκB, inhibitory kappa-B; IL, interleukin; iNOS, inducible nitric oxide synthase; LPS, lipopolysaccharide; MIA, mono-iodoacetate; MMP, matrix metalloproteinase; NF-κB, nuclear factor kappa-light-chain-enhancer of activated B cells; OA, osteoarthritis; PF, Psoraleae Fructus; PGES-2, prostaglandin synthesis E2; ROS, reactive oxygen species; RR, Rehmanniae Radix; TCM, traditional Chinese medicine; TNF-α, tumor necrosis factor-α; ZGC, Zhuanggu Guanjie capsule.
