# Supplementary figures and images for: Zhuanggu Guanjie herbal formula mitigates osteoarthritis via the NF-κB transduction mechanism
Source: Front Pharmacol. 2022 Dec 1;13:896397. doi: 10.3389/fphar.2022.896397 (PMC9751418; doi:10.3389/fphar.2022.896397)

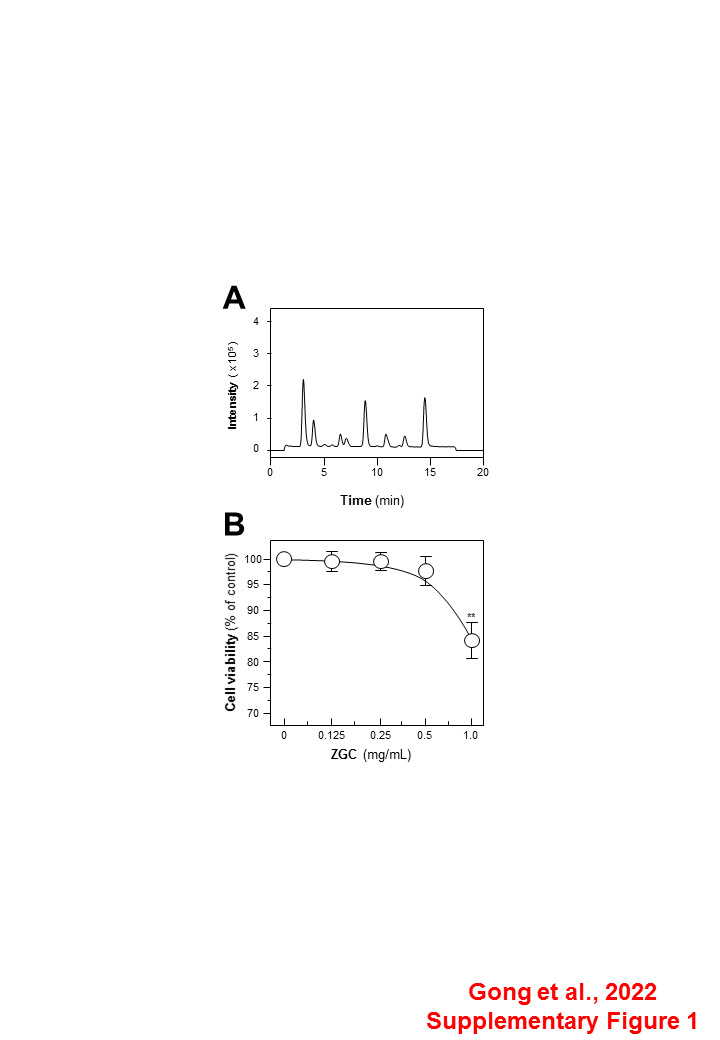

Supplement: Supplementary file 1 [file Image1.TIF]
